# Effectiveness of Stimulation of Acupoint KI 1 by *Artemisia vulgaris* (Moxa) for the Treatment of Essential Hypertension: A Systematic Review of Randomized Controlled Trials

**DOI:** 10.1155/2014/187484

**Published:** 2014-03-13

**Authors:** Xiaochen Yang, Xingjiang Xiong, Guoyan Yang, Jie Wang

**Affiliations:** ^1^Department of Cardiology, Guanganmen Hospital, China Academy of Chinese Medical Sciences, No. 5 Beixiange, Xicheng District, Beijing 100053, China; ^2^Centre for Evidence-Based Chinese Medicine, Beijing University of Chinese Medicine, Beijing 100029, China

## Abstract

*Objective*. A systematic review of randomized controlled trials has been performed to assess the effectiveness of stimulation of acupoint KI 1 by *Artemisia vulgaris* (the Japanese name is moxa) to lower blood pressure compared to antihypertensive drugs. *Methods and Findings*. Articles published from 1980 to August 2013 in databases of CENTRAL, Pubmed, CBM, CNKI, VIP, and online clinical trial registry websites were searched. Studies included were randomized controlled trials (RCTs); moxibustion-type intervention on KI 1 compared with antihypertensive drugs; meta-analysis showed superior effects of moxibustion plus antihypertensive drugs on systolic blood pressure (WMD: −4.91 [−7.54, −2.28]; *P* = 0.0003) but no superior effects on diastolic blood pressure (WMD: −6.38 [−17.17, 4.41]; *P* = 0.25). *Conclusions*. Our systematic review of the current literature shows a beneficial effect of using moxibustion interventions on KI 1 to lower blood pressure compared to antihypertensive drugs. However, the results are influenced by the existing differences in design of the current trials.

## 1. Introduction

Essential hypertension (EH) remains a major public health problem in developed and developing countries alike. It is predicted that the number of adults with hypertension will be 1.56 billion worldwide by 2025 [1]. In spite of the strength of evidence supporting the efficacy of antihypertensive agents and their wide endorsement in national and international guidelines, only about one-half of those patients are compliant with drug therapy [2]. Effective control of hypertension is limited by treatment cost, complexity, and adverse effects of antihypertensive medications [3, 4]. Perhaps, for this reason, there has been a growing interest in alternative therapies for blood pressure control [5–12].

The increasing prevalence of hypertension creates a broad market for acupuncture-type therapies to aid in the management of blood pressure [13]. The acupuncture-type therapies including moxibustion, acupuncture, electroacupuncture, and et al. are commonly used for controlling hypertension-related symptoms. Several studies have also demonstrated that acupuncture and moxibustion have good effects on the cardiovascular system, including excitation of somatic afferent input, activating sympathetic inhibitory systems in the brain related to endogenous opioids, nociceptin, *γ*-aminobutyric acid, and serotonin [14–16]. In traditional Chinese medicine, although there are many syndromes for a type of disease with complicated pathological processes, they can be classified into deficiency and excess and cold and heat. Therefore, the therapeutic principles for any disease can be summarized into four aspects, namely, supplementation, drainage, clearing, and warming.

**Figure 1:**
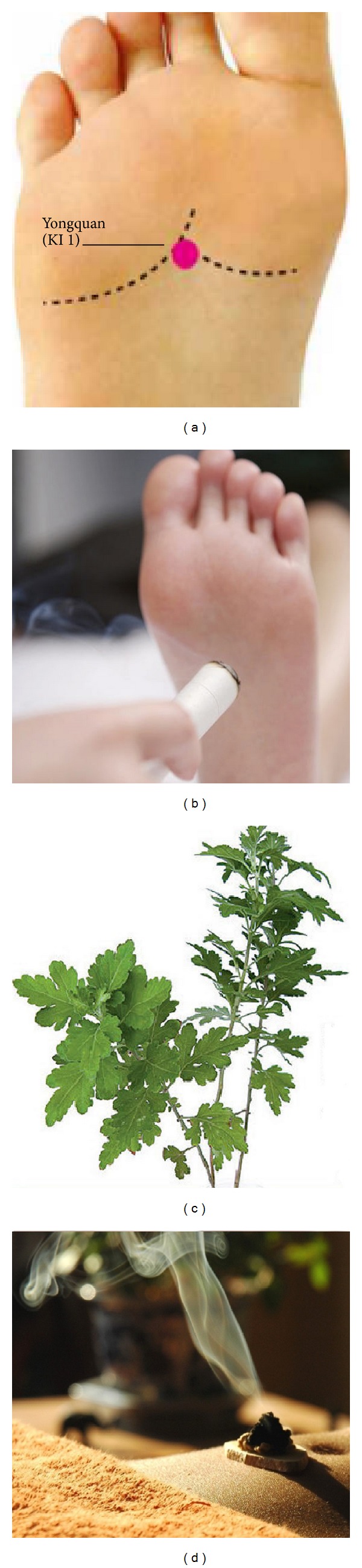
(a) Location of Yongquan, kidney 1; (b) moxibustion of Yongquan, kidney 1; (c)* Artemisia vulgaris* (the Chinese name is Ai); (d) moxibustion, a traditional Chinese method that uses the heat generated by burning herbal preparations containing* Artemisia vulgaris* to stimulate acupuncture points.

Moxibustion is another important traditional East Asian medical intervention that involves the burning of a roll of specially prepared herbs containing Artemisia vulgaris or mugwort directly or indirectly at the acupuncture points [17, 18]. KI 1 is located on the points of 1/3 and 2/3 intersection of plantar (Figure 1). In the theory of traditional Chinese medicine, KI 1 has the function of opening the orifices, directing qi downward, relieving hiccup, discharging heat, clearing heart heat, and restoring yang to save from collapse. In 1963, a cohort trial in China reported that the efficacy rate of moxibustion for the treatment of hypertension was 82.8% in the intervention group. Since this Chinese study, various studies have been performed, using acupuncture point-moxibustion, acupuncture, or electroacupuncture, to lower blood pressure [19]. In antihypertensive treatment, acupoint KI 1 is commonly used in conjunction with heat through moxibustion or electric stimulation.

The evidence examining the effectiveness of acupuncture-type interventions (moxibustion, acupuncture, and electroacupuncture) on KI 1 for essential hypertension has never been systematically summarized. The purpose of this study was to perform a systematic review of reported studies to evaluate the effectiveness of acupuncture-type interventions on KI 1 compared to conventional drugs to lower blood pressure.

## 2. Methods

### 2.1. Search Strategy

Three authors (X. Yang, X. Xiong and G. Yang) searched six electronic databases, including PubMed, the Cochrane Center Controlled Trials Register (2013), EMBASE (1980–2013), Chinese National Knowledge Infrastructure (CNKI, 1979–August 2013), Chinese Scientific Journal Database (VIP, 1989–August 2013), and Wanfang Med Online Database (WMOD, 1998–August 2013). The searching terms were “Yongquan (KI 1)”, “Gao Xue Ya Bing (essential hypertension)”, “moxibustion on KI 1 AND hypertension”, and “acupuncture on KI 1 AND hypertension”. No language restriction was applied.

### 2.2. Types of Studies

Randomized controlled trials that evaluate the effectiveness of acupuncture-type interventions (moxibustion, acupuncture, and electroacupuncture) on acupoint KI 1 for the treatment of EH were included. Quasi-RCTs were not considered.

### 2.3. Types of Participants

The participants were diagnosed as hypertensive with a systolic BP (SBP) ≥140 mm Hg and/or a diastolic BP (DBP) ≥90 mm Hg or used antihypertensive drugs. We did not intend to make any restrictions on age, gender, and race.

### 2.4. Types of Interventions

Acupuncture-type interventions (moxibustion, acupuncture, and electroacupuncture) on acupoint KI 1 were included. The study was designed to compare the effectiveness and safety of acupoint KI 1 used only or in combination with conventional drugs versus conventional drugs alone or plus placebo. All included studies should use an antihypertensive drug or no treatment as a control. Studies using interventions of unproven efficacy (e.g., herbs) in the control group were also excluded.

### 2.5. Study Selection and Data Extraction

The titles and abstracts of potentially relevant studies were identified through the literature search and reviewed independently by X. Yang and X. Xiong according to predefined criteria. Data abstraction was conducted according to predefined criteria. The following data were extracted: citations (authors of study, year of publication), methodological information, participants (sample size, age), detailed information of interventions and controls, outcome measures (systolic blood pressure, diastolic blood pressure), and adverse events. Discrepancies were discussed and resolved by consensus with other investigator (J. Wang).

### 2.6. Trial Quality Assessment and Statistical Analysis

The qualities of included RCTs were assessed by six specific domains, including random sequence generation, allocation concealment, blinding of participants and personnel, blinding of outcome data, incomplete outcome data, and selective reporting. To assess risk of bias (selection bias, performance bias, attrition bias, and reporting bias), the judgment was given as “high risk”, “unclear risk”, or “low risk”: trials that met all the criteria were categorized as low risk of bias; those that met none of the criteria were categorized as high risk of bias; and the others were categorized as unclear risk of bias if insufficient information was available to make a judgment.

According to by Cochrane Collaboration, dichotomous data were expressed as risk ratio and continuous outcomes as weighted mean difference, with their 95% confidence intervals (CI), respectively. Meta-analysis was performed if the intervention, control, and outcome were the same or similar. The statistical heterogeneity was examined with the *I* ^2^-test, where *I* ^2^ values of 50% or more were considered to be indicators of a substantial level of heterogeneity. In the absence of significant heterogeneity, we pooled data using a fixed-effect model (*I* ^2^ < 50%), otherwise we used random effects model (*I* ^2^ > 50%) [20]. To maximize the similarities among studies that would be combined, data were further stratified where possible into subgroups based on different types of interventions.

## 3. Result

### 3.1. Description of Included Trials

**Figure 2:**
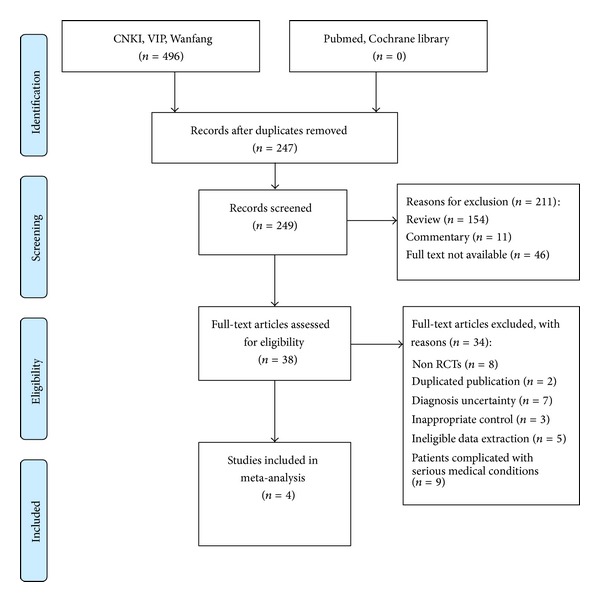
Flow diagram of the literature searching and study selection.

Our search identified 492 studies, of which we excluded 448 on the basis of the titles and abstracts and 38 studies on the basis of the full text. In total 4 RCTs were included in meta-analysis. All the RCTs were conducted in China and published in Chinese. The search for ongoing registered trials identified no trials (Figure 2).

The characteristics of the 4 RCTs that met our inclusion criteria are listed in Table 1 [21–24]. All of the included studies were conducted in China and had parallel group designs with two groups. Two RCTs [22, 23] used direct moxibustion and two [21, 24] chose indirect moxibustion treatments. The main outcome measures were the SBP or DBP in two RCTs [23, 24] and the response rate in two RCTs [21, 22].

### 3.2. Methodological Quality of Included Trials

None of the included RCTs reported any methods of sequence generation or allocation concealment. All of the included RCTs failed to report evaluator blinding. In addition, one trial mentioned drop-outs and withdrawals [21].

### 3.3. Details of Included Trials

Wang et al. [21] carried out an RCT to assess the effects of moxibustion of combined acupuncture points on blood pressure in the patients of essential hypertension. Patients were randomly divided into test group and control group. Based on the maintenance medicine treatments, 30 cases of test groups were treated by 2 months of moxibustion of combined acupuncture points. 30 cases of control groups took the maintenance medicine treatments only. Yongquan (KI 1) was used with indirect moxibustion in the patients with hypertension (*n* = 30). The primary outcome was the average value in SBP and DBP at morning, noon, and night following treatment. Moxibustion combined with drug therapy reduced the SBP and DBP significantly after 2 months of intervention compared to drug therapy only (*P* < 0.05). The changes of SBP and DBP of control groups had no statistical differences during the maintenance medicine treatment (*P* > 0.05).

Ren et al. [22] tested the effects of direct moxibustion on the BP. Sixty patients were randomized into two groups, those receiving moxibustion (2 hours, 2 times weekly for 1 month, *n* = 30) or antihypertensive drug alone (*n* = 30). The SBP and DBP were recorded before and after treatment as same as in the Wang et al. study [21]. Moxibustion combined with drug therapy reduced the SBP and DBP significantly after 10 days of intervention compared to drug therapy only (*P* < 0.05). But these parameters failed to yield significant intergroup differences.

A study performed by Jin et al. [23] consisted of an RCT evaluating the effects of moxibustion on hypertensive patients. Sixty patients were randomized into two groups, those receiving moxibustion (30 min, once a day for 10 days, *n* = 30) or antihypertensive drug alone (30 min, once a day for 10 days, *n* = 30). The outcome measures included the response rate of the reduction in BP and hypertensive symptoms including headache, dizziness, and insomnia. The response rate of the reduction in BP was referred to the percentage of responders whose SBP decreased more than 30 mmHg or whose DBP decreased more than 10 mmHg. The response rate of the reduction in hypertensive symptoms was referred to as the percentage of responders whose hypertensive symptoms according to TCM diagnosis, that is, headache, dizziness, and insomnia, decreased from the baseline levels. After treatment, the response rate of the reduction in BP was 83% in experimental group and 80% in the control group. There were no significant differences between the two groups (*P* > 0.05). 80% of the patients from the experimental group had improved hypertensive symptoms, and the corresponding rate for the control group was 67%. There were significant differences between the two groups (*P* < 0.05).

Another study [24] tested the effects of indirect moxibustion on the BP. Seventy patients were randomized into two groups, those receiving moxibustion (*n* = 50) or antihypertensive drug alone (*n* = 20). After the baseline BP measurement, indirect moxibustion was performed once in 50 patients, while nifedipine 10 mg was administered to the 20 control patients. The BP was assessed again after 30 min. The definition of the response rates in the BP and symptoms were the same as in the Jin et al. study [23]. The response rate of the BP was 70% for the moxibustion group (81% for control) and 90% for the improvement of hypertensive symptoms (control: 71%). These parameters failed to yield significant intergroup differences.

### 3.4. Effect of Interventions

**Figure 3:**
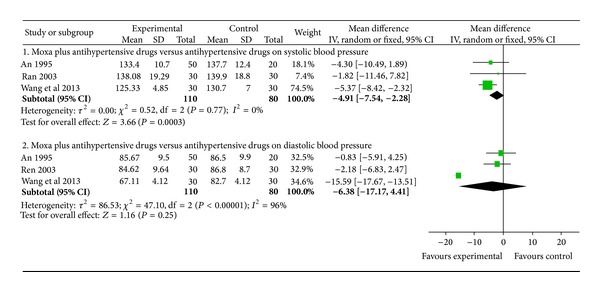
The forest plot of comparison of two groups for the outcome of systolic blood pressure and diastolic blood pressure.

In the pooled RCT's, 140 patients were included in the intervention group and 110 patients in the antihypertensive drugs (control) group (Table 1). The pooled RCT's demonstrated significant effect of the intervention: the meta-analysis showed superior effects of moxibustion plus antihypertensive drugs on systolic blood pressure (WMD: −4.91 [−7.54, −2.28]; *P* = 0.0003; Figure 3) but no superior effects on diastolic blood pressure (WMD: −6.38 [−17.17, 4.41]; *P* = 0.25; Figure 3).

### 3.5. Adverse Effect

No adverse event of direct moxibustion or indirect moxibustion on KI 1 had been mentioned among the three trials.

## 4. Discussion

In this systematic review, we found a beneficial effect of moxibustion-type interventions stimulating KI 1 to lower blood pressure compared to antihypertensive drugs. Moxibustion, like Chinese herbs, has a wide range of application. In the 1970s, WHO propagated 43 diseases that can be treated using acupuncture and moxibustion, classified into six categories: upper respiratory diseases, respiratory diseases, oral diseases, ophthalmology diseases, gastrointestinal diseases, and nervosa musculoskeletal diseases. Currently, there are more and more systematic reviews (SRs) and meta-analysis that have been conducted to assess the efficiency of acupuncture and moxibustion for EH [25–33].

The therapeutic principles of acupuncture and moxibustion are very important in guiding the formulation of treatment when treating diseases, which involves the selection of points and practical manipulation techniques. The mechanism of stimulation of KI 1 has, in part, been scientifically investigated. One study of the included trials also showed that after treatment of moxibustion on KI 1, the level of endothelin (ET), angiotensin II (AngII), plasma renin activity (PRA) decreased and nitric oxide (NO) increased but not significantly (*P* > 0.05), due to the short course of treatment. In patients with essential hypertension, structural, mechanical, or functional changes can reduce lumen diameter of arteries, which contribute to stiffer arteries.

To the best of our knowledge, this is the first systematic review and meta-analysis of RCTs for moxibustion on KI 1 in treating essential hypertension. The results showed that moxibustion on KI 1 may be effective for the treatment of essential hypertension. According to our findings of meta-analysis of SBP and DBP, moxibustion used combined with antihypertensive drugs may have good effects on patients with essential hypertension. However, this review has several limitations.

In this systematic review data from four studies were extracted, reviewed, compared, and pooled weighted mean difference was reported. A limitation of our systematic review, as with other reviews, was that the possibility of publication bias cannot be ruled out. We searched different sources to identify all RCT's and cohort studies with controls of TCM interventions on KI 1 for lowering blood pressure but there were not able to retrieve any unpublished studies. Although the use of acupuncture is widespread in China and some western countries, our literature search retrieved only four eligible trials from China and none from other countries. Databases only partially cover the literature from these countries and it is possible that unidentified eligible trials from these countries exist.

Another limitation of our study was that the low methodological quality of included trials, including the small sample sizes of RCTs and shortage of details of the study design. For example, all of the included RCTs failed to report evaluator blinding. None of the included RCTs reported any methods of sequence generation or allocation concealment. However, only one trial mentioned drop-outs and withdrawals.

Furthermore, although of the included studies the most commonly used method was moxibustion, with a protocol of once a day for 30–60 min for as long as 10–60 days, the effect of these different courses of interventions is unknown and beyond the scope of the current review. Hypertension is a chronic condition; the effect of long-term treatment is a great concern of patients. Indeed, none of the included trials reported the mortality rate or the incidence of complications. Nevertheless, not only the method of stimulation and different protocols, but also ethnic, cultural, and educational differences could account for the different results reported in the different studies.

Finally, all the included trials used blood pressure as primary outcome measure, but three of the included trials evaluated the effectiveness with numerical values. One trial presented the effect as markedly effective, effective, and ineffective. We have tried to contact authors to get further information either by telephone or email. Unfortunately, no replies and information was got. The ideal study design may be placebo controlled RCT, but due to the relative contraindication to the use of moxibustion during treatment in other locations than KI 1 it is not feasible to use a placebo moxa treatment. Besides that, a sham intervention would quickly be identifiable by the patient through information about moxibustion on the Internet.

Based on the results of our study, the effect of using moxibustion interventions on KI 1 to lower blood pressure seems promising. Our results, however, are influenced by the variety of the included studies. Therefore, before making this adjuvant treatment to standard Western healthcare, we recommend to conduct a large scale RCT in which moxibustion is compared to antihypertensive drugs following the basic guidelines for reporting clinical trials such as the Consolidated Standards of Reporting Trials (CONSORT) statement. We recommend moxibustion, as this method is inexpensive, readily available, safe, client-friendly, and can be performed at home by the partner of the hypertension patients. In addition, patients' preferences, the acceptance of the smell and warmth of the moxibustion treatment, quality of life, and healthcare and nonhealthcare costs should be assessed.

## 5. Conclusions

Our systematic review of the current literature shows a beneficial effect of using moxibustion interventions on KI 1 to lower blood pressure compared to antihypertensive drugs. Our results, however, are influenced by the existing differences in design of the current trials and further RCT's of improved quality are necessary to adequately answer this question.

## Figures and Tables

**Table 1:** Characteristics and methodological quality of included studies.

Study ID	Sample	Diagnosis standard	Intervention	Control	Course (day)	Outcome measure
Wang et al. 2013 [21]	60	CGPMHBP-2005	Moxibustion (KI1) plus metoprolol 100 mg/d	Metoprolol 100 mg/d	60	BP
Ren et al. 2003 [22]	60	1999 WHO-ISH GMH	Moxibustion (KI1) plus nifedipine controlled release tablet 20 mg/d	Nifedipine controlled release tablet 20 mg/d	30	BP
Jin et al. 2008 [23]	60	1999 WHO-ISH GMH	Moxibustion (KI1) plus enalapril 10 mg/d	Enalapril 10 mg/d	10	BP
An 1995 [24]	70	1987 WHO-ISH GMH	Moxibustion (KI1) plus nifedipine 10 mg/d	Nifedipine 10 mg/d	30	BP

Abbreviations: WHO-ISH GMH: WHO-ISH guidelines for the management of hypertension; CGPMHBP: China Guidelines on Prevention and Management of High Blood Pressure; BP: blood pressure.

## References

[1] Kearney PM, Whelton M, Reynolds K, Muntner P, Whelton PK, He J (2005). Global burden of hypertension: analysis of worldwide data. *The Lancet*.

[2] Blood Pressure Lowering Treatment Trialists’Collaboration (2003). Effects of different blood pressure lowering regimens on major cardiovascular events: second cycle of prospectively designed overviews. *The Lancet*.

[3] Shaw E, Anderson JG, Maloney M, Jay SJ, Fagan D (1995). Factors associated with noncompliance of patients taking antihypertensive medications. *Hospital Pharmacy*.

[4] Bailey JE, Lee MD, Somes GW, Graham RL (1996). Risk factors for antihypertensive medication refill failure by patients under medicaid managed care. *Clinical Therapeutics*.

[5] Wood MJ, Stewart RL, Merry H, Johnstone DE, Cox JL (2003). Use of complementary and alternative medical therapies in patients with cardiovascular disease. *American Heart Journal*.

[6] Wang J, Xiong XJ (2012). Current situation and perspectives of clinical study in integrative medicine in China. *Evidence-Based Complementary and Alternative Medicine*.

[7] Wang J, Xiong X (2012). Control strategy on hypertension in Chinese medicine. *Evidence-Based Complementary and Alternative Medicine*.

[8] Shen X, Ding G, Wei J (2006). An infrared radiation study of the biophysical characteristics of traditional moxibustion. *Complementary Therapies in Medicine*.

[9] Kim JI, Choi JY, Lee H (2010). Moxibustion for hypertension: asystematic review. *BMC Cardiovascular Disorders*.

[10] Ulbricht C, Basch E, Weissner W, Hackman D (2006). An evidence-based systematic review of herb and supplement interactions by the natural standard research collaboration. *Expert Opinion on Drug Safety*.

[11] Xiong XJ, Yang XC, Liu W, Chu FY, Wang PQ, Wang J (2013). Trends in the treatment of hypertension from the perspective of traditional Chinese medicine. *Evidence-Based Complementary and Alternative Medicine*.

[12] Wang J, Xiong XJ, Yang XC (2013). Chinese herbal medicine qi ju di huang wan for the treatment of essential hypertension: a systematic review of randomized controlled trials. *Evidence-Based Complementary and Alternative Medicine*.

[13] Ernst E, Resch KL, Mills S (1995). Complementary medicine-adefinition. *British Journal of General Practice*.

[14] Chiu YJ, Chi A, Reid IA (1997). Cardiovascular and endocrine effects of acupuncture in hypertensive patients. *Clinical and Experimental Hypertension*.

[15] Anshelevich IV, Merson MA, Afanas’eva GA (1985). Serum aldosterone level in patients with hypertension during treatment by acupuncture. *Ter Arkh*.

[16] Huang H, Liang S (1992). Acupuncture atotoacupo in the art for treatment of vascular hypertension. *Journal of Traditional Chinese Medicine*.

[17] Hitosugi N, Ohno R, Hatsukari J (2001). Diverse biological activities of moxa extract and smoke. *In Vivo*.

[18] Kobayashi K (1988). Organic components of moxa. *American Journal of Chinese Medicine*.

[19] Liao YL (1963). Effects of moxibustion of KI 1 on blood pressure in the 60 patients of essential hypertension. *Fu Jian Zhong Yi Yao*.

[20] Higgins JPT, Green S Corchrane Reviewers’Handbook 5.1.0 (updated March 2011), Review Manager (RevMan) (Computer program). Version 5.1.0.

[21] Wang R, Duan GX, Liu X (2013). Effects of moxibustion of acupuncture points on blood pressure in the patients of essential hypertension. *Medical Science Journal of Central South China*.

[22] Ren YD, Zhang X, Xia M (2003). Clinical study on treatment of essential hypertension with differentiation moxibustion. *Xin Jiang Zhong Yi Yao*.

[23] Jin RX, Liu Y, Zhao SQ (2008). Clinical study on treatment of essential hypertension with moxibustion. *Liaoning Journal of Traditional Chinese Medicine*.

[24] An SQ (1995). Clinical observation on treatment of essential hypertension with moxibustion on acupoint KI1. *Beijing Journal of Traditional Chinese Medicine*.

[25] Ernst E (2003). Cardiovascular adverse effects of herbal medicines:asystematic review of the recent literature. *Canadian Journal of Cardiology*.

[26] Bonnie J, Tesch MD (2003). Herbs commonly used by women: an evidence-based review. *American Journal of Obstetrics and Gynecology*.

[27] Xiong XJ, Yang XC, Liu YM, Zhang Y, Wang PQ, Wang J (2013). Chinese herbal formulas for treating hypertension in traditional Chinese medicine: perspective of modern science. *Hypertension Research*.

[28] Ernst E (2005). Complementary/alternative medicine for hypertension: a mini-review. *Wiener Medizinische Wochenschrift*.

[29] Wahabi HA, Alansary LA, Al-Sabban AH, Glasziuo P (2010). The effectiveness of Hibiscus sabdariffa in the treatment of hypertension: a systematic review. *Phytomedicine*.

[30] Jessica A, Leak MD (2013). Herbal medicine: is it an alternative or an unknown? A brief review of popular herbals used by patients in a pain and symptom management practice setting. *Current Review of Pain*.

[31] Wang J, Yao KW, Yang XC (2012). Chinese patent medicine liu wei di huang wan combined with antihypertensive drugs, a new integrative medicine therapy, for the treatment of essential hypertension: a systematic review of randomized controlled trials. *Evidence-Based Complementary and Alternative Medicine*.

[32] Wang J, Wang PQ, Xiong XJ (2012). Current situation and reunder standing of syndrome and formula syndrome in Chinese medicine. *Internal Medicine*.

[33] Xiong XJ, Liu W, Yang XC, Feng B, Wang J (2014). Moxibustion for essential hypertension. *Complementary Therapies in Medicine*.

